# Supplementary material for: Disentangling the role of floral sensory stimuli in pollination networks
Source: Nat Commun. 2018 Mar 12;9:1041. doi: 10.1038/s41467-018-03448-w (PMC5847531; doi:10.1038/s41467-018-03448-w)
Supplement: Supplementary file 1 — Supplementary Information [file 41467_2018_3448_MOESM1_ESM.pdf]

SUPPLEMENTARY INFORMATION

Disentangling the role of floral sensory stimuli in pollination networks

Aphrodite Kantsa, Robert A. Raguso, Adrian G. Dyer, Jens M. Olesen, Thomas Tscheulin, Theodora Petanidou

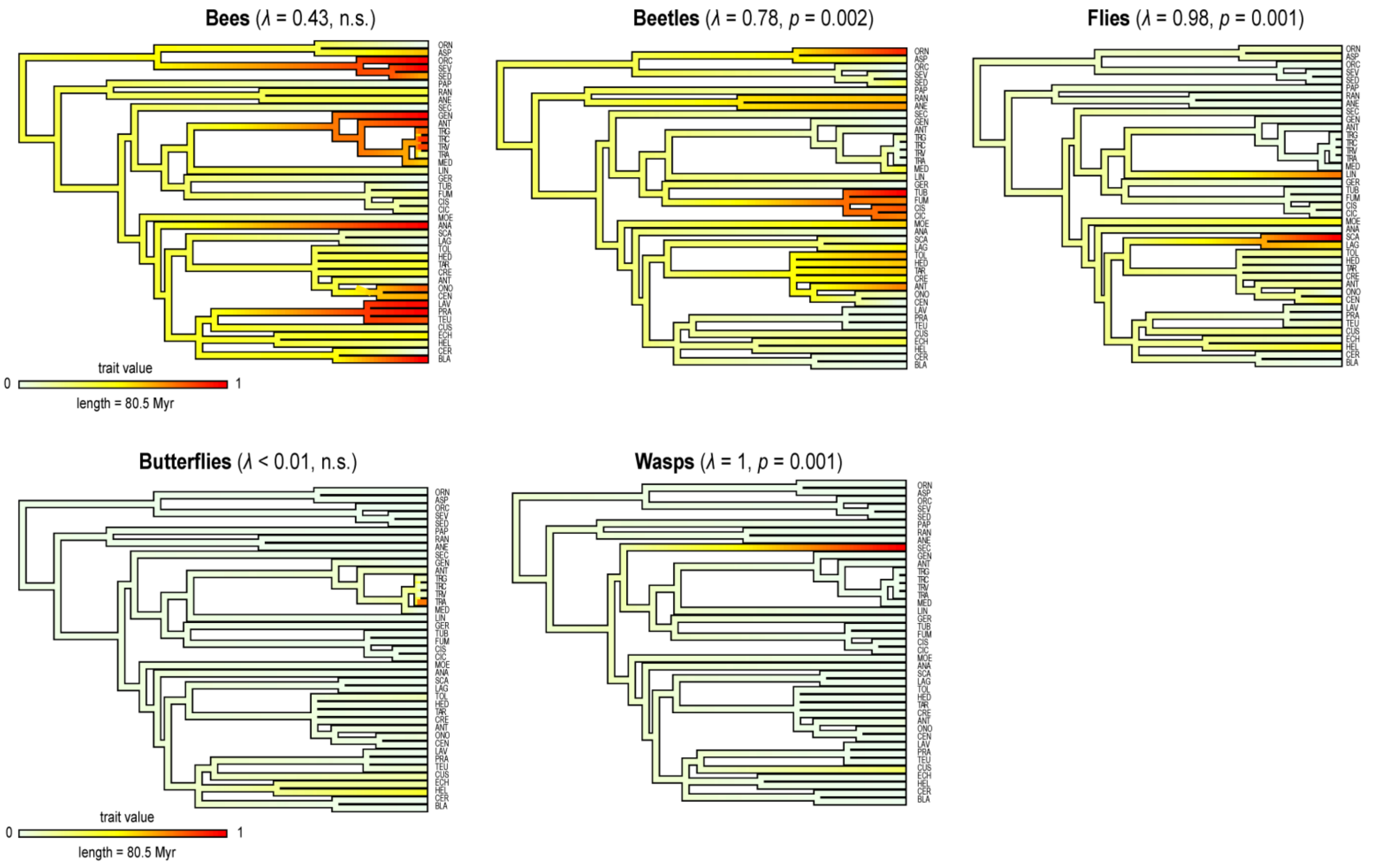

Supplementary Figure 1. The visitation rates (%) by each pollinator group mapped within the phylogram of the plants in the community. Phylogenetic signal measured with Pagel's λ is shown for every group (n.s.: $P > 0.05$) . For details, see Methods. Plant name abbreviations are explained in Table S6

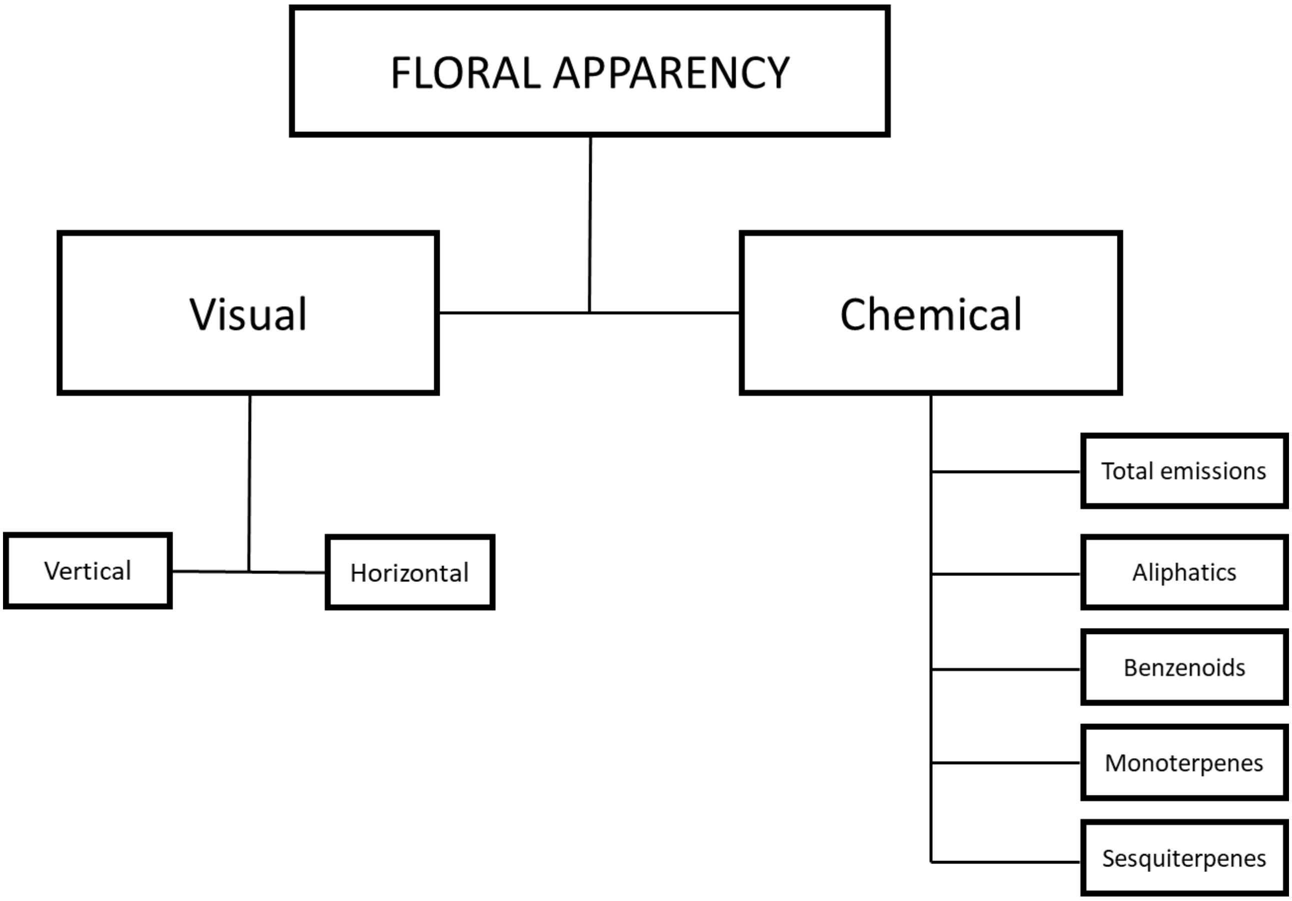

Supplementary Figure 2. The conceptual scheme including all types of floral apparency used in the present study.

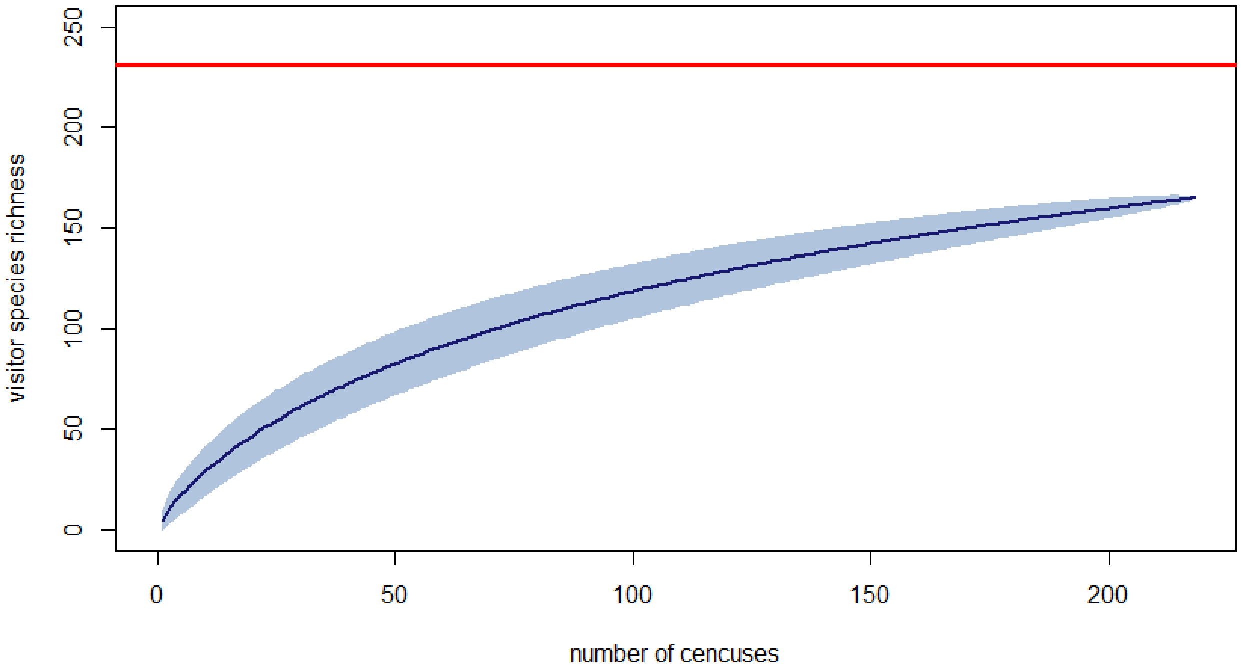

Supplementary Figure 3. Accumulation curve of the flower visitor species richness with increasing sampling effort for the phrygana network. Asymptotic species richness based on the Chao 2 estimator (red line) is shown. Sampling completeness was estimated at 73.0%.

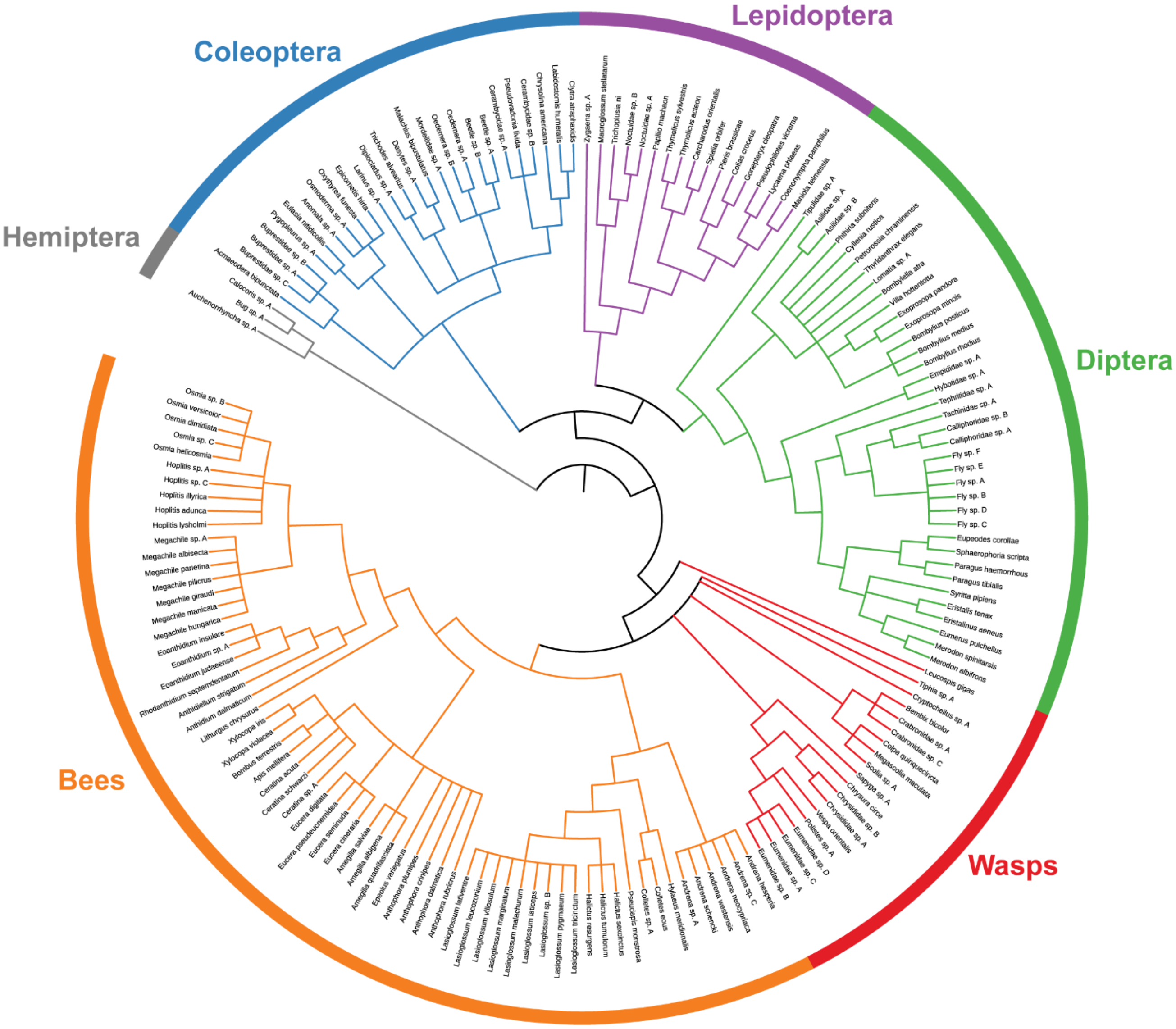

Supplementary Figure 4. The phylogeny of the flower-visiting insects in the study community. For details, see Methods.

Supplementary Table 1. The 168 flower-visiting insects recorded in the study community, and their phenology. For each Order, number of taxa is given in parentheses. Insects found only in the first year are marked with (1), and insects found only in the second year, with (2). For the rest insects, days of activity (day numbers of the Julian calendar) represent the mean values of the two years.

**Order**	**Family**	**Insect**	**First day of activity**	**Last day of activity**
Coleoptera (26; 15.5%)	Buprestidae	*Acmaeodera bipunctata*	157	173
		Buprestidae sp. A	152	155
		Buprestidae sp. B (1)	150	150
		Buprestidae sp. C (1)	157	173
	Cerambycidae	Cerambycidae sp. A	124	130
		Cerambycidae sp. B (2)	153	153
		Pseudovadonia livida	143	143
	Chrysomelidae	*Chrysolina americana* (1)	103	129
		Clytra atraphaxidis (1)	103	129
		Labidostomis humeralis (1)	105	105
	Cleridae	*Diplocladus* sp. A	147	152
		Trichodes alvearius	101	139
	Curculionidae	*Larinus* sp. A (1)	157	173
	Glaphyridae	*Eulasia nitidicollis*	117	124
		Pygopleurus sp. A	101	102
	Melyridae	*Dasytes* sp. A	103	103
		Malachius bipustulatus (1)	102	160
	Mordellidae	Mordellidae sp. A (1)	167	167
	Oedemeridae	*Oedemera* sp. A	109	166
		Oedemera sp. B	168	168
	Scarabaeidae	*Anomala* sp. A	101	143
		Epicometis hirta	101	117
		Osmoderma sp. A	130	169
		Oxythyrea funesta	133	133

Order	Family	Insect	First day of activity	Last day of activity
	Unknown	Beetle sp. A (1)	103	120
		Beetle sp. B (2)	153	153
Diptera (37; 22.0%)	Asilidae	Asilidae sp. A (1)	105	120
		Asilidae sp. B (2)	153	153
	Bombyliidae	*Bombylella atra* (1)	103	157
		Bombylius medius (1)	103	120
		Bombylius posticus	147	166
		Bombylius rhodius	101	102
		Cyllenia rustica	157	157
		Exoprosopa minois	147	161
		Exoprosopa pandora	147	176
		Lomatia sp. A (1)	150	157
		Petrorossia chraminensis (2)	153	178
		Phthiria subnitens (1)	173	173
		Thyridanthrax elegans (1)	167	167
		Villa hottentotta (2)	164	164
	Calliphoridae	Calliphoridae sp. A (1)	167	167
		Calliphoridae sp. B (1)	103	142
	Empididae	Empididae sp. A (2)	98	98
	Hybotidae	Hybotidae sp. A (1)	103	105
	Syrphidae	*Eristalinus aeneus* (2)	164	164
		Eristalis tenax	136	150
		Eumerus pulchellus	102	150
		Eupeodes corollae (2)	113	113
		Merodon albifrons (1)	150	150
		Merodon spinitarsis	121	121
		Paragus haemorrhous (1)	167	167
		Paragus sp. A (1)	173	173

Order	Family	Insect	First day of activity	Last day of activity
		Sphaerophoria scripta (1)	142	142
		Syritta pipiens (1)	167	167
	Tachinidae	Tachinidae sp. A	142	166
	Tephritidae	Tephritidae sp. A (1)	103	105
	Tipulidae	Tipulidae sp. A (1)	105	105
	Unknown	Fly sp. A	103	105
		Fly sp. B	105	105
		Fly sp. C	103	120
		Fly sp. D (1)	108	117
		Fly sp. E (1)	129	129
		Fly sp. F	105	105
Hemiptera (3; 1.8%)	Auchenorrhyncha	*Auchenorrhyncha* sp. A	142	150
	Miridae	*Calocoris* sp. A	103	105
		Bug sp. A	105	120
Hymenoptera – Bees (66; 39.3%)	Andrenidae	*Andrena* aff. *hesperia*	102	102
		Andrena aff. *neocypriaca*	128	128
		Andrena panurgimorpha (1)	135	142
		Andrena schencki (2)	113	113
		Andrena sp. A (1)	103	129
		Andrena sp. C (1)	120	129
		Andrena westensis (2)	98	178
	Apidae	*Amegilla albigena*	152	169
		Amegilla quadrifasciata	163	176
		Amegilla salviae (1)	173	173
		Anthophora crinipes (2)	113	125
		Anthophora dalmatica	109	127
		Anthophora plumipes (1)	103	103

Order	Family	Insect	First day of activity	Last day of activity
		Anthophora rubricrus (1)	105	105
		Apis mellifera	101	176
		Bombus terrestris	114	155
		Ceratina acuta (2)	125	153
		Ceratina schwarzi (2)	113	178
		Ceratina sp. A (1)	167	167
		Epeolus variegatus (1)	157	157
		Eucera cineraria group	124	143
		Eucera digitata	130	138
		Eucera aff. *pseudeucnemidea* (2)	125	125
		Eucera seminuda	139	139
		Xylocopa iris	114	169
		Xylocopa violacea	141	166
	Colletidae	*Colletes eous* (2)	136	136
		Colletes sp. A	147	147
		Hylaeus meridionalis (1)	167	167
	Halictidae	*Halictus resurgens*	147	176
		Halictus sexcinctus (1)	167	173
		Halictus aff. *tumulorum.confusus* (2)	153	153
		Lasioglossum laticeps/mediterraneum (2)	136	153
		Lasioglossum aff. *lativentre/haesitans* (2)	136	136
		Lasioglossum leucozonium	109	155
		Lasioglossum malachurum group	152	157
		Lasioglossum marginatum (1)	129	129
		Lasioglossum pygmaeum/pauperatum	101	133
		Lasioglossum sp. B (2)	113	113
		Lasioglossum tricinctum group	101	160
		Lasioglossum villosulum group (2)	136	136

Order	Family	Insect	First day of activity	Last day of activity
		Pseudapis monstrosa (2)	136	153
	Megachilidae	*Anthidiellum strigatum*	155	155
		Anthidium aff. *dalmaticum* (2)	153	153
		Eoanthidium insulare (2)	178	178
		Eoanthidium aff. *judaense* (2)	160	160
		Eoanthidium sp. A	164	164
		Hoplitis adunca group (2)	125	125
		Hoplitis aff. *illyrica*	141	141
		Hoplitis aff. *lysholmi*	152	155
		Hoplitis sp. A (2)	125	125
		Hoplitis sp. C (1)	142	142
		Lithurgus chrysurus (1)	173	173
		Megachile albisecta	163	163
		Megachile giraudi (2)	136	136
		Megachile hungarica	147	173
		Megachile manicata (2)	98	153
		Megachile parietina (2)	98	136
		Megachile pilicrus	155	176
		Megachile sp. A (1)	167	167
		Osmia dimidiata	163	163
		Osmia aff. *helicosmia* (2)	125	125
		Osmia sp. B (2)	125	125
		Osmia sp. C (2)	125	125
		Osmia versicolor (2)	125	125
		Rhodanthidium septemdentatum (2)	125	178
Hymenoptera – Wasps (19; 11.3%)	Chrysididae	*Chrysididae* sp. A (1)	157	157
		Chrysididae sp. B (2)	113	113
		Chrysura circe (1)	103	103

Order	Family	Insect	First day of activity	Last day of activity
	Crabronidae	*Bembix bicolor* (2)	136	153
		Crabronidae sp. A (1)	150	150
		Crabronidae sp. C (2)	164	178
	Eumenidae	Eumenidae sp. A (1)	167	167
		Eumenidae sp. B (2)	136	136
		Eumenidae sp. C (2)	136	136
		Eumenidae sp. D (2)	136	136
	Leucospidae	*Leucospis gigas* (1)	167	167
	Pompilidae	*Cryptocheilus* sp. A	117	124
	Sapygidae	*Sapyga* sp. A (1)	135	135
	Scoliidae	*Colpa quinquecincta* (2)	178	178
		Megascolia maculata	147	155
		Scolia sp. A (2)	164	178
	Tiphiidae	*Tiphia* sp. A (2)	113	113
	Vespidae	*Polistes* sp. A	164	173
		Vespa orientalis (1)	142	167
Lepidoptera (17; 10.1%)	Hesperiidae	*Charcharodus orientalis* (2)	153	178
		Spialia orbifer (2)	178	178
		Thymelicus acteon (1)	157	157
		Thymelicus sylvestris	160	160
	Lycaenidae	*Lycaena phlaeas*	143	161
		Pseudophilotes vicrama	128	155
	Noctuidae	Noctuidae sp. A (1)	167	167
		Noctuidae sp. B (2)	136	136
		Trichoplusia ni (1)	157	157
	Nymphalidae	*Coenonympha pamphilus*	141	157
		Maniola telmessia	130	143
	Papilionidae	*Papilio machaon* (1)	157	167

Order	Family	Insect	First day of activity	Last day of activity
	Pieridae	*Colias crocea*	128	155
		Gonepteryx cleoptatra (2)	136	136
		Pieris brassicae (1)	157	167
	Sphingidae	*Macroglossum stellatarum* (2)	136	153
	Zygaenidae	Zygaenidae sp. A (2)	125	125

Supplementary Table 2. Module composition of the entire network in the study community. For details, see Methods.

Module		Species
#1	Plants	*Sedum confertiflorum*
	Insects	*Chrysididae* sp. *B*
		Chrysura circe
#2	Plants	*Anthemis auriculata*
		Blackstonia perfoliata
		Heliotropium lasiocarpum
		Linum trigynum
		Trifolium arvense
	Insects	*Bombylius posticus*
		Cerambycidae sp. *A*
		Ceratina schwarzi
		Charcharodus orientalis
		Chrysididae sp. *A*
		Cyllenia rustica
		Eucera digitata
		Eupeodes corollae
		Exoprosopa minois
		Exoprosopa pandora
		Hoplitis illyrica aff.
		Hoplitis lysholmi aff.
		Lasioglossum lativentre/haesitans aff.
		Sapyga sp. *A*
		Spialia orbifer
#3	Plants	*Anagallis arvensis*
		Cistus creticus
		Lavandula stoechas subsp. *stoechas*
		Prasium majus
		Serapias cordigera
		Trifolium angustifolium
	Insects	*Acmaeodera bipunctata*
		Anthophora crinipes
		Anthophora dalmatica
		Anthophora plumipes
		Anthophora rubricrus
		Asilidae sp. *A*
		Beetle sp. *B*
		Bembix bicolor
		Bombylius medius
		Cerambycidae sp. *B*
		Chrysolina americana
		Crabronidae sp. *A*
		Cryptocheilus sp. *A*
		Eoanthidium insulare
		Eristalinus aeneus
		Eristalis tenax

Module		Species
		Eucera cineraria-group
		Eucera seminuda
		Eumenidae sp. *B*
		Eumenidae sp. *C*
		Hoplitis sp. *A*
		Lasioglossum laticeps/mediterraneum
		Lasioglossum pygmaeum/pauperatum
		Maniola telmessia
		Merodon albifrons
		Oedemera sp. *B*
		Pseudapis monstrosa
		Pseudovadonia livida
		Rhodanthidium septemdentatum
		Tipulidae sp. *A*
		Villa hottentotta
		Xylocopa iris
		Zygaenidae sp. *A*
**#4**	Plants	*Cuscuta epithymum*
	Insects	*Asilidae* sp. *B*
		Calliphoridae sp. *A*
		Colletes sp. *A*
		Colpa quinquecincta
		Crabronidae sp. *C*
		Eoanthidium judaense aff.
		Eoanthidium sp. *A*
		Eumenidae sp. *A*
		Hylaeus meridionalis
		Leucospis gigas
		Megascolia maculata
		Mordellidae sp. *A*
		Paragus haemorrhous
		Paragus sp. *A*
		Polistes sp. *A*
		Scolia sp. *A*
		Syritta pipiens
		Thyridanthrax elegans
**#5**	Plants	*Asphodelus ramosus*
		Moenchia mantica
		Scandix sp.
	Insects	*Andrena* sp. *A*
		Bombylius rhodius
		Calocoris sp. *A*
		Empididae sp. *A*
		Eumerus pulchellus
		Fly sp. *A*
		Fly sp. *B*
		Fly sp. *C*
		Fly sp. *D*
		Fly sp. *F*

Module		Species
		Hybotidae cf. sp. *A*
		Tephritidae sp. *A*
		Trichodes alvearius
**#6**	Plants	*Anthyllis hermanniae*
		Echium plantagineum
		Genista acanthoclada
		Orchis sancta
	Insects	*Anthidiellum strigatum*
		Ceratina acuta
		Ceratina sp. *A*
		Colletes eous
		Epeolus variegatus
		Hoplitis adunca-group
		Lasioglossum leucozonium
		Lasioglossum sp. *B*
		Lycaena phlaeas
		Megachile manicata
		Megachile parietina
		Noctuidae sp. *B*
		Osmia helicosmia aff.
		Oxythyrea funesta
		Pseudophilotes vicrama
**#7**	Plants	*Centaurea solstitialis*
		Onopordum tauricum
		Teucrium divaricatum
	Insects	*Amegilla albigena*
		Amegilla quadrifasciata
		Amegilla salviae
		Anthidium dalmaticum aff.
		Bombus terrestris
		Buprestidae sp. *A*
		Buprestidae sp. *C*
		Colias crocea
		Diplocladus sp. *A*
		Gonepteryx cleoptatra
		Halictus resurgens
		Halictus sexcinctus
		Larinus sp. *A*
		Lithurgus chrysurus
		Macroglossum stellatarum
		Megachile albisecta
		Megachile giraudi
		Megachile hungarica
		Megachile pilicrus
		Megachile sp. *A*
		Noctuidae sp. *A*
		Osmia dimidiata
		Papilio machaon
		Phthiria subnitens

Module		Species
		Pieris brassicae
		Osmoderma sp. *A*
		Thymelicus acteon
		Thymelicus sylvestris
		Trichoplusia ni
		Vespa orientalis
		Xylocopa violacea
**#8**	Plants	*Anemone pavonina*
		Cistus salvifolius
		Fumana arabica
		Hedypnois cretica
		Lagoecia cuminoides
		Medicago minima
		Ornithogalum sp.
		Ranunculus paludosus
		Taraxacum sp.
		Tolpis barbata
		Trifolium campestre
		Trifolium glanduliferum var. *nervulosum*
		Tuberaria guttata
	Insects	*Andrena hesperia* aff.
		Andrena schencki
		Andrena westensis
		Anomala cf. sp. *A*
		Apis mellifera
		Auchenorrhyncha sp. *A*
		Beetle sp. *A*
		Bombylella atra
		Bug sp. *A*
		Coenonympha pamphilus
		Dasytes sp. *A*
		Epicometis hirta
		Eulasia nitidicollis
		Eumenidae sp. *D*
		Labidostomis humeralis
		Lasioglossum tricinctum (group)
		Lomatia sp. *A*
		Malachius bipustulatus
		Oedemera sp. *A*
		Osmia sp. *B*
		Osmia versicolor
		Pygopleurus sp. *A*
		Tachinidae sp. *A*
**#9**	Plants	*Crepis neglecta*
		Serapias vomeracea
	Insects	*Andrena neocypriaca* aff.
		Andrena panurgimorpha
		Andrena sp. *C*
		Buprestidae sp. *B*

**Module**	**Species**
	Calliphoridae sp. *B*
	Clytra atraphaxidis
	Eucera pseudeucnemidea aff.
	Fly sp. *E*
	Halictus tumulorum/confusus aff.
	Hoplitis sp. *C*
	Lasioglossum malachurum (group)
	Lasioglossum marginatum
	Lasioglossum villosulum-group
	Merodon spinitarsis
	Osmia sp. *C*
	Petrorossia chraminensis
	Sphaerophoria scripta
	Tiphia sp. *A*

Supplementary Table 3. Sizes of the *phenonets* in the study community, connectance and nestedness. Metrics are also shown for the *phenonets* of the plants that received no visits.

phenonet	**Connectance (%)**	**Nestedness (%)**	**# Insects**	**# Plants**
Anagallis arvensis	6.7	95.2	158	37
Anemone pavonina	13.9	82.5	47	24
Anthemis auriculata	6.6	95.3	165	36
Anthyllis hermanniae	16.5	85.4	51	20
Asphodelus ramosus	14.4	81.8	47	23
Blackstonia perfoliata	9.2	92.8	68	36
Centaurea solstitialis	19	82.2	42	16
Centaurium pulchellum	19.8	80.5	42	15
Cistus creticus	6.7	95.2	158	37
Cistus salviifolius	8.3	92.9	99	34
Crepis neglecta	7.8	94.6	115	35
Cuscuta epithymum	14.3	86.6	77	16
Echium plantagineum	6.8	95.8	143	37
Fumana arabica	6.7	95.2	158	37
Genista acanthoclada	13.7	82.5	47	24
Geranium robertianum	8.9	94.4	91	32
Hedypnois cretica	8.5	93.4	96	33
Heliotropium lasiocarpum	NA	NA	NA	NA
Lagoecia cuminoides	11.4	91.1	68	27
Lavandula stoechas	7.1	94.3	139	37
Linum trigynum	6.8	95.8	143	37
Medicago minima	15.2	89.9	46	18
Moenchia mantica	7.2	94.0	135	37
Onopordum tauricum	13.5	87.0	88	16
Orchis sancta	9.9	89.7	72	31
Ornithogalum sp.	17.1	78.7	30	24
Papaver argemone	11.4	92.5	49	28
Prasium majus	14	89.4	41	25
Ranunculus paludosus	15.5	79.5	39	19
Scandix sp.	9.9	89.7	72	31
Sedum confertiflorum	13.8	82.6	48	24
Serapias cordigera	9	92.6	83	31
Serapias vomeracea	9.8	89.8	72	30
Taraxacum sp.	12.4	83.5	54	27
Teucrium divaricatum	11.8	90.8	99	20
Tolpis barbata	7.1	95.7	136	35
Trifolium angustifolium	11.8	88.9	50	29
Trifolium arvense	8.2	94.2	109	33
Trifolium campestre	10.8	85.1	67	28
Trifolium glanduliferum	11.3	85.7	64	27
Tuberaria guttata	9.9	89.7	72	31

Supplementary Table 4. Comparison of the node properties computed within the 'static' network of the entire flowering season and for the plant *phenonets* in the community. Properties were calculated using the function 'specieslevel' in the R package *bipartite* v.2.05, except for the functional role which was computed with the *NetCarto* software (see Methods). Plants with no insect visitors (*viz. Centaurium pulchellum, Geranium robertianum,* and *Papaver argemone*) as well as *Heliotropium lasiocarpum* that flowered alone, are not included. For details on each property see Materials and Methods.

	Normalized Degree		Betweenness Centrality		Closeness Centrality		Functional Role	
**Plant**	**static**	***phenonet***	**static**	***phenonet***	**static**	***phenonet***	**static**	***phenonet***
Anagallis arvensis	0.006	0.006	0.000	0.000	0.020	0.021	Peripheral	Peripheral
Anemone cf. *pavonina*	0.024	0.085	0.007	0.020	0.028	0.043	Peripheral	Connector
Anthemis auriculata	0.071	0.073	0.062	0.052	0.031	0.032	Connector	Connector
Anthyllis hermanniae	0.071	0.137	0.025	0.040	0.029	0.050	Module hub	Peripheral
Asphodelus ramosus	0.065	0.213	0.008	0.034	0.027	0.045	Peripheral	Peripheral
Blackstonia perfoliata	0.006	0.015	0.000	0.000	0.021	0.023	Peripheral	Peripheral
Centaurea solstitialis	0.018	0.048	0.004	0.015	0.023	0.052	Peripheral	Peripheral
Cistus creticus	0.411	0.430	0.207	0.194	0.035	0.035	Network hub	Network hub
Cistus salviifolius	0.113	0.182	0.020	0.025	0.031	0.035	Peripheral	Connector
Crepis neglecta	0.155	0.200	0.124	0.138	0.030	0.032	Module hub	Module hub
Cuscuta epithymum	0.143	0.260	0.021	0.114	0.030	0.068	Module hub	Connector
Echium plantagineum	0.054	0.063	0.025	0.027	0.030	0.030	Peripheral	Peripheral
Fumana arabica	0.077	0.082	0.059	0.066	0.032	0.033	Connector	Connector
Genista acanthoclada	0.012	0.043	0.000	0.000	0.020	0.029	Peripheral	Peripheral
Hedypnois cretica	0.054	0.094	0.013	0.017	0.031	0.035	Peripheral	Peripheral
Lagoecia cuminoides	0.012	0.029	0.001	0.002	0.027	0.036	Peripheral	Peripheral
Lavandula stoechas	0.125	0.137	0.102	0.107	0.032	0.032	Connector	Peripheral
Linum trigynum	0.048	0.056	0.032	0.025	0.030	0.030	Peripheral	Module hub
Medicago minima	0.018	0.065	0.014	0.047	0.029	0.058	Peripheral	Peripheral
Moenchia mantica	0.065	0.074	0.009	0.010	0.029	0.029	Connector	Connector
Onopordum tauricum	0.208	0.375	0.033	0.049	0.029	0.059	Module hub	Module hub
Orchis sancta	0.012	0.028	0.008	0.021	0.026	0.032	Peripheral	Peripheral
Ornithogalum sp.	0.030	0.100	0.004	0.009	0.028	0.043	Peripheral	Peripheral
Prasium majus	0.024	0.073	0.000	0.000	0.020	0.029	Peripheral	Peripheral
Ranunculus paludosus	0.024	0.103	0.004	0.011	0.028	0.055	Peripheral	Peripheral
Scandix sp.	0.065	0.153	0.001	0.002	0.023	0.029	Module hub	Module hub
Sedum confertiflorum	0.012	0.042	0.000	0.000	0.000	0.000	Peripheral	Peripheral
Serapias cordigera	0.018	0.036	0.001	0.001	0.021	0.025	Peripheral	Peripheral
Serapias vomeracea	0.006	0.014	0.000	0.000	0.016	0.021	Peripheral	Peripheral
Taraxacum sp.	0.089	0.278	0.046	0.117	0.031	0.044	Peripheral	Connector
Teucrium divaricatum	0.119	0.172	0.050	0.106	0.030	0.050	Peripheral	Module hub
Tolpis barbata	0.077	0.081	0.047	0.029	0.032	0.033	Peripheral	Peripheral
Trifolium angustifolium	0.006	0.020	0.000	0.000	0.019	0.025	Peripheral	Peripheral
Trifolium arvense	0.060	0.092	0.050	0.055	0.032	0.036	Connector	Peripheral
Trifolium campestre	0.006	0.015	0.000	0.000	0.025	0.034	Peripheral	Peripheral
Trifolium glanduliferum	0.054	0.094	0.021	0.025	0.031	0.042	Peripheral	Peripheral
Tuberaria guttata	0.018	0.042	0.002	0.002	0.027	0.034	Peripheral	Peripheral

Supplementary Table 5. Vertical, horizontal, and chemical floral apparency in the study community.

Plant	Vertical apparency	Horizontal apparency	Total emissions apparency	Aliphatics apparency	Benzenoids apparency	Monoterpenes apparency	Sesquiterpenes apparency
Anagallis arvensis	-12.89	0.50	-0.15	0.18	0.09	-0.09	0.00
Anemone pavonina	4.91	1.44	-0.45	-0.10	0.00	-0.04	0.00
Anthemis auriculata	-2.80	2.26	0.50	0.13	0.01	0.36	0.30
Anthyllis hermanniae	11.56	-0.37	80.94	1.69	12.61	63.25	1.78
Asphodelus ramosus	58.94	0.75	-0.73	-0.29	-0.01	-0.10	0.00
Blackstonia perfoliata	-8.59	-0.96	-0.52	-0.07	-0.01	-0.09	0.00
Centaurea solstitialis	6.98	-1.62	-1.12	-0.24	-0.01	-0.24	-0.05
Centaurium pulchellum	-9.77	-1.82	-1.43	-0.29	-0.02	-0.34	-0.09
Cistus creticus	16.86	139.73	19.56	0.31	1.97	4.63	8.63
Cistus salvifolius	14.28	64.10	2.55	0.81	0.26	0.15	0.98
Crepis neglecta	11.40	8.41	0.90	0.77	0.00	0.14	0.00
Cuscuta epithymum	8.35	-0.96	-0.86	-0.12	0.05	-0.23	-0.05
Echium plantagineum	-9.38	-0.54	-0.57	-0.14	0.00	-0.08	0.00
Fumana arabica	4.15	2.83	-0.11	0.16	0.04	-0.02	0.00
Genista acanthoclada	24.86	-1.12	0.24	-0.13	0.45	0.15	0.06
Geranium robertianum	-12.21	-1.25	0.61	-0.23	0.01	0.80	0.38
Hedypnois cretica	-5.79	-0.14	0.73	0.53	0.07	0.21	-0.01
Lagoecia cuminoides	0.19	-0.26	26.36	-0.23	3.08	23.07	0.83
Lavandula stoechas	17.68	137.13	391.45	4.96	0.00	350.03	30.70
Linum trigynum	-5.69	0.98	-0.26	-0.16	0.00	0.26	0.00
Medicago minima	-10.32	-1.54	-1.33	-0.39	-0.01	-0.31	-0.08
Moenchia mantica	-3.38	2.22	1.21	1.50	0.00	-0.05	0.00
Onopordum tauricum	92.94	14.19	-1.15	-0.24	-0.01	-0.27	-0.05
Orchis sancta	2.72	-1.18	2.74	-0.20	3.25	-0.12	0.09
Ornithogalum sp.	-9.06	-1.16	-0.64	-0.28	-0.01	-0.07	-0.01
Papaver argemone	0.48	-0.72	-0.94	-0.27	-0.01	-0.18	-0.02
Prasium majus	46.57	-1.26	-0.72	-0.26	-0.01	-0.07	0.02

**Plant**	**Vertical apparency**	**Horizontal apparency**	**Total emissions apparency**	**Aliphatics apparency**	**Benzenoids apparency**	**Monoterpenes apparency**	**Sesquiterpenes apparency**
Ranunculus paludosus	-4.00	-1.51	-0.25	-0.28	-0.02	0.43	-0.01
Scandix sp.	2.41	2.01	219.88	2.47	214.56	2.09	1.13
Sedum confertiflorum	-10.98	-1.62	-0.67	-0.28	0.00	-0.07	0.01
Serapias cordigera	3.36	3.68	0.14	0.64	-0.01	-0.11	0.00
Serapias vomeracea	3.69	-0.15	0.19	0.65	-0.01	-0.10	0.00
Taraxacum sp.	8.90	0.78	-0.69	-0.24	-0.01	-0.11	-0.01
Teucrium divaricatum	25.87	4.34	24.66	4.00	-0.01	6.28	14.79
Tolpis barbata	-1.87	4.76	0.06	0.26	0.00	-0.05	0.05
Trifolium angustifolium	-9.29	-1.27	-0.36	0.06	0.00	-0.09	-0.01
Trifolium arvense	-8.73	6.66	-0.27	-0.21	0.03	0.10	-0.01
Trifolium campestre	-7.53	1.47	2.83	2.46	0.34	0.30	0.02
Trifolium glanduliferum	-9.23	6.60	80.12	6.60	64.84	5.80	2.02
Tuberaria guttata	-0.54	0.35	-0.28	-0.14	-0.01	0.22	-0.01

Supplementary Table 6. Flowering phenology, floral morphology and the volatility of the scent blends of the plants in the community. Days of flowering and floral density represent mean values of the two years of sampling, except for *Geranium robertianum* and *Heliotropium europaeum* that were observed in the community only in the second year. Note that *Cuscuta epithymum* is a parasitic vine that was found on *Cistus creticus* towards the end of its flowering period.

Abbrv.	Plant	First day of flowering[1]	Last day of flowering[1]	Floral density (fl. units/m^2)	Corolla depth[2]	Symmetry	Floral surface (cm^2)	Floral height (cm)	Mean nBP (^oC)
ANA	*Anagallis arvensis*	102	169	3.23	short	actinomorphic	0.47	3.71	218.1
ANE	*Anemone pavonina*	102	117	0.23	short	actinomorphic	13.67	20.67	222.9
ANL	*Anthemis auriculata*	117	169	0.67	short	actinomorphic	4.95	13.97	207.7
ANT	*Anthyllis hermanniae*	141	152	8.58	short	zygomorphic	0.12	28.79	227.5
ASP	*Asphodelus ramosus*	102	117	0.71	short	actinomorphic	3.39	74.65	206.7
BLA	*Blackstonia perfoliata*	117	138	1.09	short	actinomorphic	0.29	6.74	188.8
CEP	*Centaurea solstitialis*	152	155	0.01	long	actinomorphic	0.58	25.95	213.1
CES	*Centaurium pulchellum*	147	152	0.01	short	actinomorphic	0.49	10.11	228.5
CIC	*Cistus creticus*	102	173	8.29	short	actinomorphic	16.97	33.48	235.2
CIS	*Cistus salvifolius*	102	146	6.70	short	actinomorphic	9.74	30.28	209.6
CRE	*Crepis neglecta*	117	161	3.69	long	actinomorphic	2.56	28.20	205.6
CUS	*Cuscuta epithymum*	152	173	1.50	short	actinomorphic	0.47	27.35	221.4
ECH	*Echium plantagineum*	110	169	0.32	long	zygomorphic	1.39	7.25	169.9
FUM	*Fumana arabica*	102	173	0.96	short	actinomorphic	3.97	20.75	206.3
GEN	*Genista acanthoclada*	102	117	0.73	long	zygomorphic	0.73	40.53	208.2
GER	*Geranium robertianum*	125	153	0.08	short	actinomorphic	0.56	4.63	226.8
HED	*Hedypnois cretica*	102	143	1.99	long	actinomorphic	0.44	9.82	202.2
HEL	*Heliotropium lasiocarpum*	178	178	0.10	short	actinomorphic	4.42	16.71	200.9
LAG	*Lagoecia cuminoides*	130	152	1.30	short	actinomorphic	0.66	17.21	180.5
LAV	*Lavandula stoechas*	102	163	28.99	long	zygomorphic	4.76	34.23	209.1
LIN	*Linum trigynum*	110	169	18.05	short	actinomorphic	0.11	10.87	166.0
MED	*Medicago minima*	136	146	0.56	short	zygomorphic	0.07	5.10	201.2
MOE	*Moenchia mantica*	102	157	3.34	long	actinomorphic	0.96	13.33	222.1
ONO	*Onopordum tauricum*	147	176	0.40	long	actinomorphic	39.43	111.47	202.5

Abbrv.	Plant	First day of flowering[1]	Last day of flowering[1]	Floral density (fl. units/m^2)	Corolla depth[2]	Symmetry	Floral surface (cm^2)	Floral height (cm)	Mean nBP (°C)
ORC	*Orchis sancta*	102	130	0.07	long	zygomorphic	1.01	18.33	261.8
ORN	*Ornithogalum* sp.	117	122	0.12	short	actinomorphic	3.44	7.00	194.8
PAP	*Papaver argemone*	128	141	0.02	short	actinomorphic	19.81	16.31	244.6
PRA	*Prasium majus*	122	133	0.04	long	zygomorphic	1.35	61.75	205.6
RAN	*Ranunculus paludosus*	102	110	0.16	short	actinomorphic	2.35	13.70	198.2
SCA	*Scandix* sp.	102	132	3.01	short	actinomorphic	1.08	18.00	237.8
SED	*Sedum confertiflorum*	102	117	0.54	short	actinomorphic	0.12	4.66	211.1
SEC	*Serapias cordigera*	102	138	1.86	long	zygomorphic	2.60	18.68	268.3
SEV	*Serapias vomeracea*	102	130	1.43	long	zygomorphic	0.72	19.56	274.5
TAR	*Taraxacum* sp.	102	125	0.20	long	actinomorphic	10.77	24.25	247.0
TEU	*Teucrium divaricatum*	139	176	7.80	long	zygomorphic	0.74	43.50	215.1
TOL	*Tolpis barbata*	117	173	2.93	short	actinomorphic	1.98	14.96	212.1
TRA	*Trifolium angustifolium*	117	130	1.94	short	zygomorphic	0.03	6.55	202.9
TRV	*Trifolium arvense*	122	161	7.66	short	zygomorphic	1.00	7.75	200.1
TRC	*Trifolium campestre*	102	128	3.48	short	zygomorphic	0.80	7.95	206.8
TRG	*Trifolium glanduliferum*	102	127	6.54	short	zygomorphic	1.22	5.99	217.7
TUB	*Tuberaria guttata*	102	134	1.02	short	actinomorphic	1.54	15.12	202.3

[1]Day numbers of the Julian calendar.
[2]short < 3 mm; long ≥ 3 mm.

Supplementary Table 7. The phylogenetic signal in all floral traits and attributes used in the present study. Different methods have been used according to variable type (see Methods). *D:* statistic for binary traits; P_{rand}: probability of *D* resulting from no (random) phylogenetic structure; P_{Brown}: probability of *D* resulting from Brownian phylogenetic structure. DM: distance matrix (Bray-Curtis). *P*-values implying significance ($P \leq 0.050$) are shown in bold.

Trait/matrix	Pagel's λ	*P*	*D*	P_{random}	$P_{Brownian}$	Mantel r	*P*
Trichromatic saturation: r_{tri}	0.22	0.199					
Trichromatic hue: θ_{tri}	0.00	1.000					
Tetrachromatic saturation: r_{tet}	**0.83**	**0.003**					
Tetrachromatic hue angle: φ_{tet}	0.00	1.000					
Tetrachromatic hue angle: θ_{tet}	0.00	1.000					
Proportion (%) of aliphatics	0.00	1.000					
Proportion (%) of benzenoids	0.00	1.000					
Proportion (%) of monoterpenes	0.00	1.000					
Proportion (%) of sesquiterpenes	0.38	0.232					
Apparency of aliphatic emissions	0.00	1.000					
Apparency of benzenoid emissions	0.00	1.000					
Apparency of monoterpene emissions	0.00	1.000					
Apparency of sesquiterpene emissions	0.21	0.675					
Apparency of total emissions	0.00	1.000					
Normal boiling point of the scent blend	**0.87**	**0.008**					
Floral height apparency	0.00	1.000					
Floral surface apparency	0.00	1.000					
Start date	0.20	0.497					
Flowering duration	0.00	1.000					
Normalized degree	0.00	1.000					
Betweenness centrality	**0.47**	**0.043**					
Closeness centrality	0.00	1.000					
Nectar			0.09	**0.006**	**0.471**		
Symmetry			-0.75	**0.000**	**0.955**		
Corolla depth			-0.15	**0.000**	**0.958**		
Plant interactions DM (weighted)						0.09	0.062
Pollinator interactions DM (weighted)						-0.01	0.671

Supplementary Table 8. The independent variales (predictors) used in the present study in order to estimate the best models (PGLS or MGLM) according to AIC.

Variable	Type
Trichromatic saturation: r_{tri}	Continuous
Trichromatic hue: θ_{tri}	Continuous
Trichromatic color: $r_{tri} \times \theta_{tri}$	Interaction term – Continuous
Tetrachromatic saturation: r_{tet}	Continuous
Tetrachromatic hue: θ_{tet} and φ_{tet}	Continuous
Tetrachromatic color: $r_{tet} \times \theta_{tet} \times \varphi_{tet}$	Interaction term – Continuous
Proportion (%) of aliphatics in the scent blend	Proportion
Proportion (%) of benzenoids in the scent blend	Proportion
Proportion (%) of monoterpenes in the scent blend	Proportion
Proportion (%) of sesquiterpenes in the scent blend	Proportion
Apparency of aliphatic emissions	Continuous
Apparency of benzenoid emissions	Continuous
Apparency of monoterpene emissions	Continuous
Apparency of sesquiterpene emissions	Continuous
Interaction of VOC class apparencies	Interaction term – Continuous
Apparency of total emissions	Continuous
Normal boiling point of the scent blend	Continuous
Nectar presence/absence	Binary
Symmetry (actinomorphic/zygomorphic)	Binary
Corolla depth (short/long)	Binary
Floral height apparency	Continuous
Floral surface apparency	Continuous
Flowering phenology (start date × flowering duration)	Interaction term – Integers
