# Supplementary material for: Disentangling the role of floral sensory stimuli in pollination networks
Source: Nat Commun. 2018 Mar 12;9:1041. doi: 10.1038/s41467-018-03448-w (PMC5847531; doi:10.1038/s41467-018-03448-w)
Supplement: Supplementary file 3 — Description of Additional Supplementary Files [file 41467_2018_3448_MOESM3_ESM.pdf]

Description of Supplementary Files

File Name: Supplementary Data 1
Description: The plant-pollinator network of the study scrubland in Lesvos Island, Greece.

File Name: Supplementary Data 2
Description: Inflorescence volatile compounds of the plant species studied.
